# Characterization of Antifungal Natural Products Isolated from Endophytic Fungi of Finger Millet (*Eleusine* *coracana*)

**DOI:** 10.3390/molecules21091171

**Published:** 2016-09-03

**Authors:** Walaa Kamel Mousa, Adrian L. Schwan, Manish N. Raizada

**Affiliations:** 1 Department of Plant Agriculture, University of Guelph, Guelph, ON N1G 2W1, Canada; wmoatey@uoguelph.ca; 2 Department of Pharmacognosy, Mansoura University, Mansoura 35516, Egypt; 3 Department of Chemistry, University of Guelph, Guelph, ON N1G 2W1, Canada; schwan@uoguelph.ca

**Keywords:** finger millet, *Fusarium* sp., endophyte, fungus, *Penicillium* sp., 5-hydroxy 2(3*H*)-benzofuranone, dehydrocostus lactone, harpagoside

## Abstract

Finger millet is an ancient African-Indian crop that is resistant to many pathogens including the fungus, *Fusarium* *graminearum*. We previously reported the first isolation of putative fungal endophytes from finger millet and showed that the crude extracts of four strains had anti-*Fusarium* activity. However, active compounds were isolated from only one strain. The objectives of this study were to confirm the endophytic lifestyle of the three remaining anti-*Fusarium* isolates, to identify the major underlying antifungal compounds, and to initially characterize the mode(s) of action of each compound. Results of confocal microscopy and a plant disease assay were consistent with the three fungal strains behaving as endophytes. Using bio-assay guided fractionation and spectroscopic structural elucidation, three anti-*Fusarium* secondary metabolites were purified and characterized. These molecules were not previously reported to derive from fungi nor have antifungal activity. The purified antifungal compounds were: 5-hydroxy 2(3H)-benzofuranone, dehydrocostus lactone (guaianolide sesquiterpene lactone), and harpagoside (an iridoide glycoside). Light microscopy and vitality staining were used to visualize the in vitro interactions between each compound and *Fusarium*; the results suggested a mixed fungicidal/fungistatic mode of action. We conclude that finger millet possesses fungal endophytes that can synthesize anti-fungal compounds not previously reported as bio-fungicides against *F.* *graminearum*.

## 1. Introduction

Finger millet (*Eleusine* *coracana*) is an ancient cereal crop widely grown by subsistence farmers in Africa and India. Finger millet was domesticated in Ethiopia and Uganda at 5000 BC and then reached India by 3000 BC [1]. Unlike other related cereals, finger millet is well known for its ability to tolerate stress conditions and to resist many pathogens, including *Fusarium* *graminearum* [2,3,4,5]. *F. graminearum* is a serious fungal pathogen that causes Fusarium Head Blight (FHB) in wheat and Gibberella Ear Rot (GER) in corn [6]. Both diseases result in catastrophic losses in grain yield and are associated with mycotoxin accumulation in grains [7]. *Fusarium* sp. are ancient fungi that have been dated to 8.8 mya in Africa [8], the same continent where finger millet was domesticated.

We hypothesized that the potential long-term co-evolution between finger millet and *Fusarium* sp. may not only have placed selection pressure on the genome of finger millet but also on its associated microbiome. Specifically, we hypothesized that finger millet may host endophytes that confer resistance to *F. graminearum*. Endophytes have been reported to help their host plants to combat pathogens [2,9,10].

**Figure 1:**
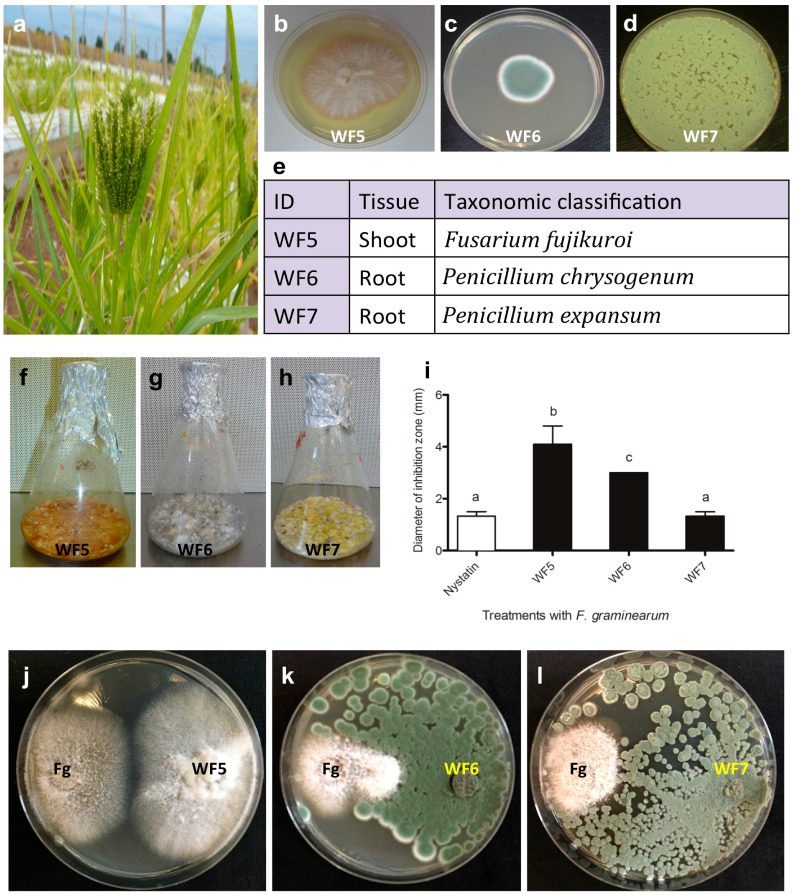
Characterization of the putative fungal endophytes previously isolated from finger millet and the antifungal activity of their extracts. (**a**) Picture of a finger millet plant; (**b**–**d**) Pictures of fungal endophytes WF4, WF5 and WF7 isolated from finger millet, respectively; (**e**) Predicted fungal endophyte nomenclatures, alongside the corresponding plant tissue from which they were isolated and best BLAST match taxonomic identification based on 18S rDNA sequencing; (**f**–**h**) Example of each endophyte fermented on rice medium as indicated; (**i**) Quantification of the effects of each endophyte extract (250 mg/mL) on the growth of *F.* *graminearum* compared to the fungicide nystatin (10 µg/mL) (diameter of inhibition zone in mm, *n* = 3); (**j**–**l**) Representative images illustrating the results of co-incubation of each endophytic fungus with *F.* *graminearum* in vitro.

Consistent with our hypothesis, in a previous study, we reported the isolation of four putative fungal endophytes (strains WF4-7) from finger millet and showed that their extracts had anti-fungal activity against *F. graminearum* [2]. Only one of these endophytes, *Phoma* sp. strain WF4, was analyzed in detail including isolation of the underlying anti-*Fusarium* compounds. Of the remaining strains, WF6 and WF7 (*Penicillium* sp.) were isolated from finger millet roots, while WF5 (*Fusarium* sp.) was isolated from shoots (Figure 1) [2]. The crude culture extracts of each strain inhibited the growth of *F.* *graminearum* (Figure 1) [2]. The objectives of this study were to confirm the endophytic behaviour of the three remaining, putative, anti-*Fusarium* fungal endophytes, to identify the underlying major antifungal compounds and to study the mode of action of each pure compound.

## 2. Results

### 2.1. Confirming the Endophytic Behaviour of the Putative Fungal Endophytes

To confirm the endophytic behaviour of the isolated finger millet fungi, two experiments were undertaken.

#### 2.1.1. Plant Pathogenicity Assay

**Figure 2:**
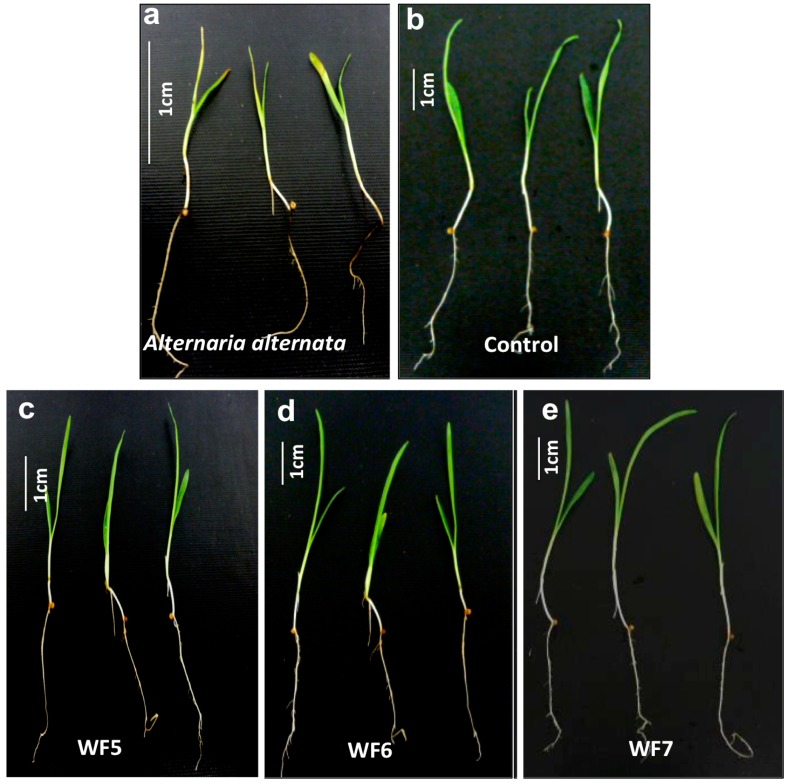

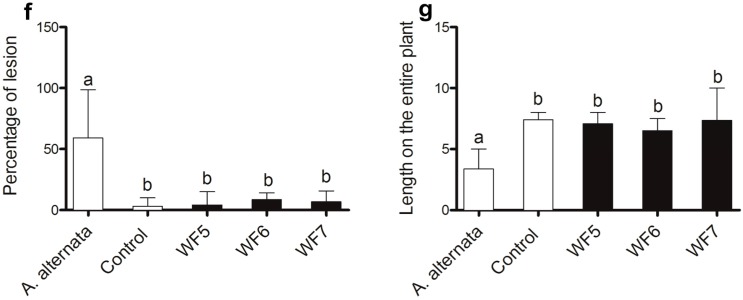
Plant pathogenicity assay of each putative endophyte following inoculation onto finger millet. (**a**) Representative picture showing the effect of the known pathogen *Alternaria* *alternata* on finger millet seedlings (positive control); (**b**) Representative picture showing the effect of buffer on finger millet seedlings (negative control); (**c**–**e**) Representative pictures showing the effect of the each putative fungal endophyte on finger millet seedlings as indicated; (**f**) Quantification of the percentage of lesions caused by each fungus or the pathogen *Alternaria* *alternata* compared to the control using Assess software; (**g**) Quantification of the total length of finger millet seedlings inoculated with each endophytic fungus or the pathogen *Alternaria* *alternata* compared to the control. For both (**f**) and (**g**), letters that are different from one another indicate that their means are statistically different (Mann-Whitney *t*-test, *p* ≤ 0.05). The whiskers indicate the standard error of the mean.

Seedlings of finger millet were co-incubated with each putative fungal endophyte or with a known pathogen of finger millet, *Alternaria* *alternata*. The seedlings inoculated with the pathogen developed disease symptoms including black roots and leaf spots and reduction in plant length (Figure 2a), compared to control seedlings that received the buffer only (Figure 2b). However, none of the putative fungal endophytes showed statistically significant disease symptoms on finger millet seedlings compared to the pathogen control (Figure 2c–g).

#### 2.1.2. Root Colonization Assay

**Figure 3:**
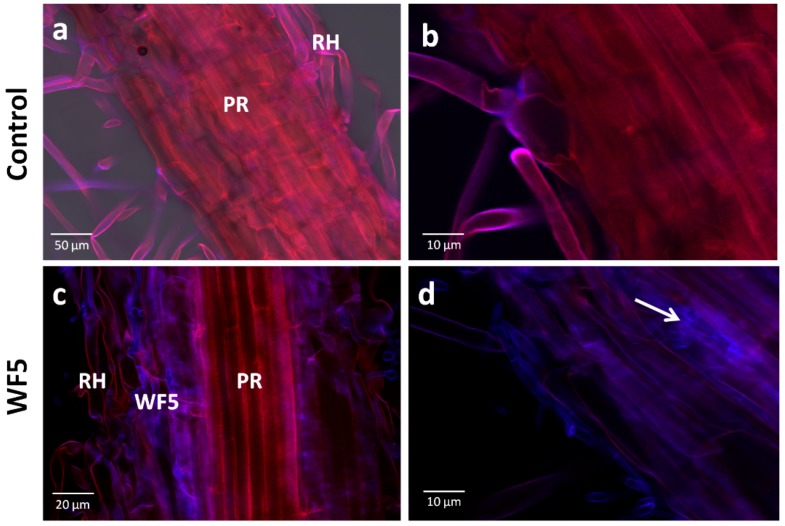

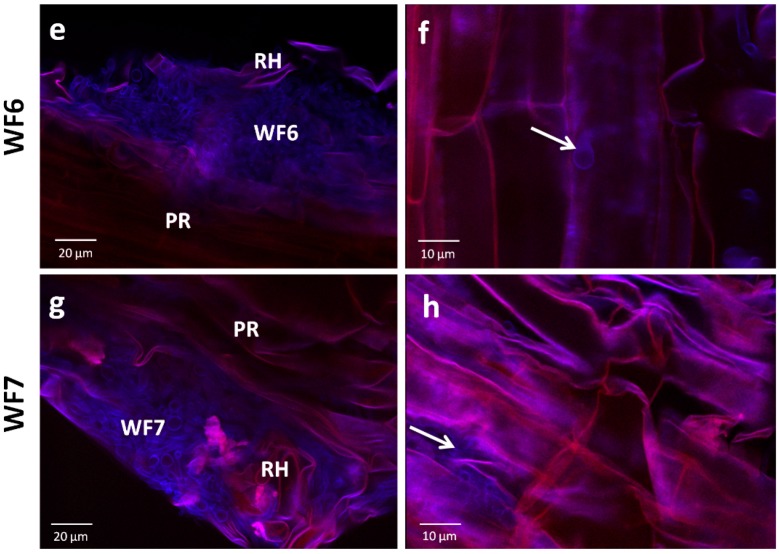
Test for the ability of the putative fungal endophytes to colonize finger millet roots using confocal scanning laser microscopy. (**a**–**b**) Representative pictures of root tissues inoculated with the buffer control; (**c**–**h**) Representative pictures of root tissues inoculated with each putative fungal endophyte as indicated. Fungi fluoresce purple-blue due to staining with calcofluor. Plant tissues appear red due to auto-fluorescence. White arrows point to fungi inside the tissues. Abbreviations: PR, primary root; RH, root hair.

To test the ability of each endophyte to colonize the internal tissues of finger millet roots, confocal microscopy imaging was conducted. Seedlings inoculated with the buffer only (control) showed no observable fungal growth inside the tissues (Figure 3a,b). At the early post-inoculation time point used, most of the fungal hyphae were observed on the rhizoplane, however each of the fungi was visualized to initiate colonization of the epidermal and sub-epidermal layers of finger millet roots, providing further support that they are endophytes of finger millet (Figure 3c–h).

### 2.2. Bio-Assay Guided Purification and Structural Elucidation of Anti-Fusarium Compounds

**Figure 4:**
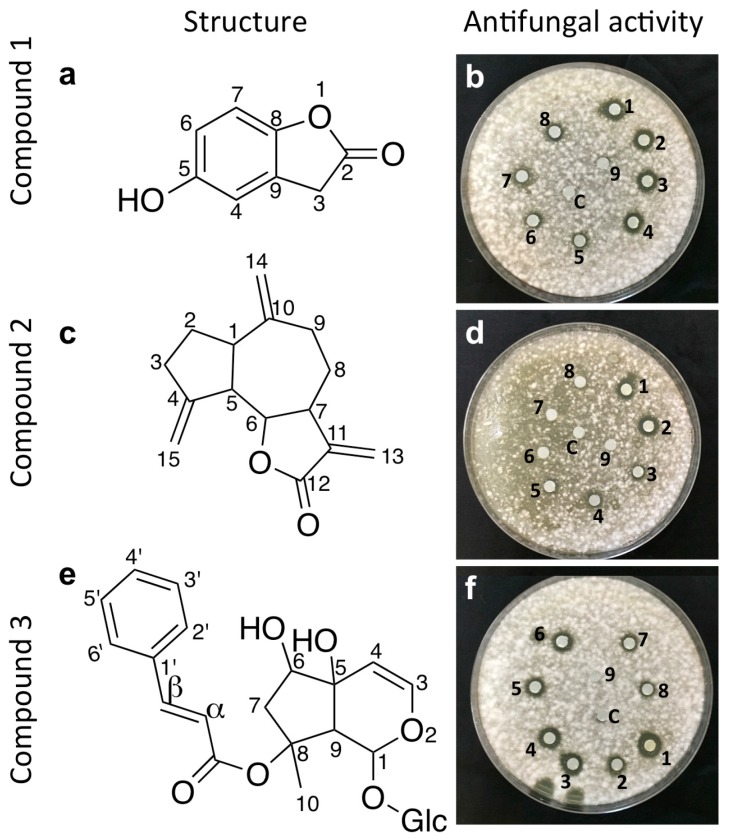
Structures and in vitro activity of the purified anti-*Fusarium* compounds from fungal endophyte strains WF5, WF6 and WF7. (**a**) Structure of compound 1,5-hydroxy benzofuranone (from strain WF5); (**b**) Representative picture of the disc diffusion assay showing the anti-*Fusarium* activity of compound **1**; (**c**) Structure of compound **2**, dehydrocostus lactone (from strain WF6); (**d**) Representative picture of the disc diffusion assay showing the anti-*Fusarium* activity of compound **2**; (**e**) Structure of compound **3**, harpagoside (from strain WF7); (**f**) Representative picture of the disc diffusion assay showing the anti-*Fusarium* activity of compound **3**. Numbers (1–9) denote a concentration gradient of 4000, 2000, 1000, 500, 250, 125, 62.5, 31.25, 15.62 and 7.8 μg/mL while C denotes the solvent control.

Bio-assay guided purification was conducted to isolate the active anti-fungal compounds from each endophyte extract. Three active anti-*Fusarium* compounds were purified (Figure 4). The diameter of zones of growth inhibition of *F.* *graminearum* were 2, 2.5 and 1.5 cm for compounds **1**, **2** and **3**, respectively. The minimum inhibitory concentration (MIC) for compounds **1**–**3** were 31.25, 250.00 and 31.25 µg/mL, respectively. The three compounds were subjected to further spectroscopic structure elucidation. Results corresponding to each compound are shown separately below. 1D- and 2D-NMR spectra for these three compounds are presented (Figures S1–S15).

### 2.3. Structural Elucidation of the Isolated Antifungal Compounds

#### 2.3.1. Compound **1** (Isolated from Fungus WF5)

Compound **1** (molecular formula C_8_H_6_O_3_) was eluted with hexane-ethyl acetate (90:10) as a white amorphous powder, with an Rf value of 0.46, and further moved as a single band in hexane-ethyl acetate (85:15), with an Rf of 0.58. The yield of compound **1** was 50 mg from 3 g of total extract. IR: 3320, 1762, 1604, 1241, 1077, 948 cm^−1^. ^1^H-NMR (600 MHz, DMSO): 3.83 (2H, s, H-3), 6.65 (1H, dd, *J* = 8.6, 1.6 Hz, H-6), 6.75 (1H, d, *J* = 1.6 Hz, H-4), 6.95 (1H, d, *J* = 8.6 Hz, H-7), 9.32 (1H, s, Ar–OH). ^13^C-NMR (150 MHz, DMSO): 175 (C-2), 153.9 (C-8), 146.7 (C-5), 125.1 (C-9), 114.2 (C-4), 111.8 (C-6), 110.5 (C-7), 33.3 (C-3). Comparing spectral data (Figures S1–S5) with a previous reference [11], the compound was confirmed as 5-hydroxy 2(3*H*)-benzofuranone (Figure 4a).

#### 2.3.2. Compound **2** (Isolated from Fungus WF6)

Compound **2** (molecular formula C_15_H_18_O_2_) was eluted from the hexane-ethyl acetate (50:50) fraction as a colorless solid. The compound was then purified by preparative TLC using a solvent mixture of hexane-ethyl acetate (30:70), with an Rf value of 0.73. The yield of compound **2** was 10 mg from 3 g total extract. The IR and 1D-NMR data were as follows: IR: 2934, 1765, 1257, 1146, 998 cm^−1^. ^1^H-NMR (600 MHz, CDCl_3_): δ 2.91 (1H, m, H-1), 1.91 (1H, m, H-2 α), 1.85 (1H, m, H-2β), 2.14 (1H, m, H-3α), 2.5 (2H, m, H-3), 2.87 (1H, m, H-5), 3.95 (1H, t, *J* = 10 Hz, H6), 2.90 (1H, m, H-7), 2.24 (1H, m, H-8α), 1.42 (1H, m, H-8β), 2.48 (1H, m, H-9α), 2.19 (1H, m, H-9β), 6.22 (1H, d, *J* = 3.6 Hz, H-13α), 5.47 (1H, d, *J* = 3.6, H-13β), 4.87 (1H, s, H-14α), 4,79 (1H, s, H-14β), 5.25 (1H, d, *J* = 2.4 Hz, H-15α), 5.04 (1H, d, *J* = 2.4 Hz, H-15β). ^13^C-NMR (150 MHz, CDCl_3_): δ 30.2 (C-3), 30.9 (C-8), 32.4 (C-2), 36.2 (C-9), 45.09 (C-1), 47.5 (C-7), 52.0 (C-5), 85.2 (C-6), 109.59 (C-14), 112.6 (C-15), 120.19 (C-13), 139.7 (C-11), 149.2 (C-10), 151.2 (C-4), 170.27(C-12). Comparing spectral data (Figures S6–S10) with reference data [12], the compound was confirmed as dehydrocostus lactone, an guaianolide sesquiterpene lactone (Figure 4c).

#### 2.3.3. Compound **3** (Isolated from Fungus WF7)

Compound **3** (molecular formula C_24_H_30_O_11_) was eluted from the 100% methanol fraction as an amorphous powder. The compound was further purified using preparative TLC with a mobile phase mixture of methanol-water (95:5), with an Rf value of 0.12. The yield of compound **3** was 5 mg from 3 g total extract. The IR and 1D-NMR data were as follows: IR: 3392, 2884, 1334, 1283, 989, 770 cm^−1^. ^1^H-NMR (600 MHz, CDCl_3_): 1.33 (3H, s, H-10), 1.74 (1H, dd, *J* = 14.5 and 4 Hz, H-7α), 2.15 (1H, d, *J* = 14.5 Hz, H-7β), 2.93 (1H, s, H-9), 3.36-3.84 (Glc-2-6), 4.6 (Glc-1), 3.98 (1H, d, *J* = 4 Hz, H-6), 4.83 (1H, d, *J* = 8 Hz, H-4), 6.1 (1H, s, H-1), 6.36 (1H, d, *J* = 6.4 Hz, H-3), 6.38 (1H, d, *J* = 16 Hz, H-α), 7.47 (3H, m, H3′-5′), 7.55 (2H, m, H-2′ and 6′), 7.58 (1H, d, *J* = 16 Hz, H- β). ^13^C-NMR (150 MHz, CDCl_3_): 94(C-1), 143.4 (C-3), 104.7 (C-4), 72.7 (C- 5), 76.5 (C-6), 45.0 (C-7), 87.3 (C-8), 53.4 (C-9), 22.4 (C-10), 119 (C-α), 144.9 (C-β), 167.3 (C=O), 134.2 (C-1′), 128.8 (C-2′), 128.2 (C-3′), 130.3 (C-4′), 128.2 (C-5′), 128.8(C-6′), 99.1 (Glc-1′), 72.7 (Glc-2), 75.9 (Glc-3), 71.70 (Glc-4), 75.9 (Glc-5), 61.8 (Glc-6). Comparing spectral data (Figures S11–S15) results to the literature [13], the compound was identified as harpagoside, an iridoide glycoside (Figure 4e).

### 2.4. Microscopic Examination of the Interaction Between Each Pure Antifungal Compound and F. graminearum *in Vitro*

**Figure 5:**
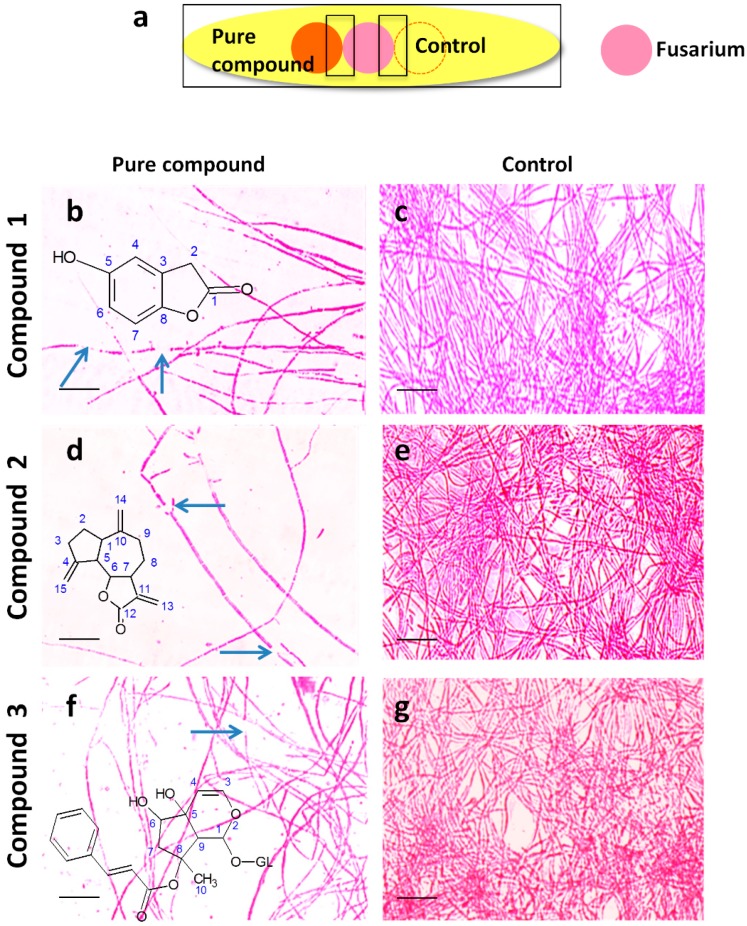
The effects of the purified endophyte-derived anti-fungal compounds on *F.* *graminearum* in vitro using neutral red staining. (**a**) Cartoon of the experimental methodology used to examine the in vitro interactions between *F.* *graminearum* (pink) and each compound (orange) or the buffer control (respective compound solvent). Microscope slides were pre-coated with PDA and incubated for 24 h. *F.* *graminearum* hyphae were then stained with neutral red. Shown are representative microscope slide pictures (*n* = 3) of the interactions of *F.* *graminearum* with: (**b**) 5-hydroxy benzofuranone (5 mg/mL) compared to (**c**) the buffer control; (**d**) dehydrocostus lactone (5 mg/mL) compared to (**e**) the buffer control; (**f**) harpagoside (5 mg/mL) compared to (**g**) the buffer control. The blue arrows point to areas of apparent breakage of *F.* *graminearum* hyphae. Each scale bar equals 25 µm.

To visualize the interaction between each pure compound and *F.* *graminearum* in vitro, light microscopy was used (Figure 5).

All compounds resulted in reduced hyphal growth associated with frequent hyphal breakage (Figure 5b,d,f), compared to the control (Figure 5c,e,g), suggestive of a mixed fungistatic/fungicidal mode of action. Interestingly, the hyphae of *F. graminearum* appeared to bend away from the contact zone with compounds **2** and **3** (Figure 5d,f). However, the effect of all purified compounds on *F.* *graminearum* hyphae was weak/moderate when compared to other antifungal compounds previously isolated from finger miller fungal endophytes [2].

## 3. Discussion

Endophytes are defined as microbes that are able to colonize the internal tissues of their host plants without causing disease [14]. We hypothesized that finger millet may host endophytes with anti-fungal activity, including against the pathogen *Fusarium* *graminearum*, because this crop is known to be resistant to many pathogens including *F.* *graminearum* [2,3,5], and its endophytes may have co-evolved with *Fusarium* pathogens in Africa [15,16] and South Asia [17,18]. In a previous study [2], we identified four distinct, putative, fungal endophyte species from finger millet (WF4-7). The putative endophytes were isolated from plants of first generation seeds, and then grown on Turface clay rock using hydroponics rather than on soil. This growth system may have contributed to the low abundance and biodiversity of the isolated endophytes.

In our earlier study [2], we showed that extracts of the putative endophytes have antifungal activities including against *F.* *graminearum*. Only the extract from WF4, a *Phoma* sp. was characterized in detail, and revealed four anti-*Fusarium* compounds (viridicatol, tenuazonic acid, alteraniol and alteraniol methyl ether). Here, we confirmed that these fungal strains are able to re-colonize the internal tissues of the host without causing pathogenic symptoms, when compared to the known pathogen *Alternaria* *alternate*, consistent with their classification as endophytes. We identified the compounds underlying the antifungal activity of these endophytes alongside a suggested mode of action. The current anti-*Fusarium* compounds are different than those previously identified and broaden the range of bio-fungicides identified from the finger millet microbiome against this important crop pathogen.

### 3.1. Previous Reports of Non-Pathogenic Fusarium and Penicillium sp. as Endophytes

One fungal strain reported in this study was predicted to be a *Fusarium* sp. (WF5). Previous studies involving *Fusarium* and *Aspergillus* sp. have shown that non-pathogenic fungal strains that belong to the same species as a pathogen can, in some cases, control the ability of the pathogen to cause disease and produce mycotoxins [19,20]. Several competitive exclusion mechanisms have been suggested that explain the biological control ability [21]. These mechanisms include blockage of infection sites, competition for limited nutrients in the soil, inhibition of spore germination and induction of host resistance [22,23]. In the current study, we showed that the ability of non-pathogenic *Fusarium* sp. to control pathogenic *Fusarium* sp. may also be mediated by production of antifungal secondary metabolites.

The two other fungal strains (WF6 and WF7) characterized in this study were closely related to *Penicillium* sp., including one that most closely resembled *Penicillium* *chrysogenum* (WF6) taxonomically. *P.* *chrysogenum* was previously isolated as an endophyte of marine red algal species of the genus *Laurencia*, and was reported to have antifungal activity against the fungus *Alternaria* *brassicae.* The antifungal metabolite was identified to be a mono-terpene derivative [24]. This observation is of interest because the anti-*Fusarium* compound that we purified from the apparent *P.* *chrysogenum* isolate from finger millet is a sesquiterpene lactone, suggesting that the terpenoid pathway may play an important role in the anti-pathogenesis of *P.* *chrysogenum* endophytes. Other *Penicillium* sp. were isolated from diverse hosts including the South Asian medicinal plant *Ocimum* *tenuiflorum* [25], the Moroccan plant *Ceratonia* *siliqua* [26], *Cannabis* *sativa* L. [27] and *Panax* *ginseng* [28]. An extract from the *Penicillium* isolate of *Panax ginseng* showed anti-fungal activity against *Pyricularia* *oryzae* [28]. We emphasize this last observation because *P.* *oryzae* is the most important fungal pathogen of finger millet, the causal agent of blast disease [29].

### 3.2. Anti-Fusarium Compounds Purified from Finger Millet Endophytes

Here, we have elucidated the structures of three active anti-*Fusarium* compounds: 5-hydroxy benzofuranone, dehydrocostus lactone, and an iridoide glycoside (harpagoside) (Figure 4).

#### 3.2.1. 4-Hydroxybenzofuranone (Isolated from *Fusarium* sp. Strain WF5)

Simple benzofuranone derivatives have previously been isolated from fungi including *Coniothyrium* minitans [30]. Other conjugated benzofuranones have been reported from endophytic fungi such as *Pestalotiopsis* *photiniae*, an endophyte that inhabits the Chinese plant, *Roystonea* *regia* [31]. Some benzofuranone derivatives were reported to have algicidal, hypotensive and anti-inflammatory activities [32,33]. To the best of our knowledge, this is the first report of 4-hydroxy- benzofuranone having anti-*Fusarium* activity.

#### 3.2.2. Dehydrocostus Lactone (Isolated from *Penicillium* sp. Strain WF6)

Dehydrocostus lactone derivatives were previously isolated from plant species including *Inula* *racemosa,* *Centaurea* *pannonica* and *Saussurea* *costus* [34,35,36]. The compound was reported to have antimicrobial activity including against *Mycobacterium* sp., *Propionibacterium* *acnes*, *Staphylococcus* *aureus*, and *Malassezia* *furfur* [36,37]. Dehydrocostus lactone was reported to have multiple biological activities including suppression of melanin production, induction of apoptosis in soft tissue sarcoma cell lines, inhibition of migration of prostate cancer cells and anti-trypanosoma activity [35,36,38,39]. To the best of our knowledge, this is the first report of dehydrocostus lactone from a fungal source and the first report of it having anti-*Fusarium* activity.

#### 3.2.3. Harpagoside (Isolated from *Penicillium* sp. Strain WF7)

Harpagoside was first isolated from *Harpagophytum* *procumbens* (Devil’s claw), a plant widely used in folk medicine for its anti-microbial, anti-inflammatory and analgesic properties [40]. Subsequently, harpagoside was identified in diverse plants including *Radix* *scrophulariae*, a plant used as an herbal medicine for treatment of congestion, constipation and sore throats [41]. Harpagoside was reported to have anti-inflammatory, antioxidant and analgesic properties [42] To the best of our knowledge, this is the first report of dehydrocostus lactone from a fungal source and the first report of it having anti-*Fusarium* activity.

## 4. Experimental Section

### 4.1. Source of Biological Materials

Putative fungal endophyte strain WF5 (*Fusarium* sp.) was previously from finger millet shoots (Genbank: KF957640) using a previously described protocol (Figure 1) [2]. Putative fungal endophyte strains WF6 and WF7 (*Penicillium* sp.) were previously isolated from finger millet roots (Genbank: KF957641 and KF957642) (Figure 1) [2]. In brief, commercial finger millet seeds originating from India were germinated and grown in pails of Turface clay placed in the field (Arkell Field Station, Arkell, ON, Canada, GPS: 43°39′ N, 80°25′ W, and 375 m above sea level). At the pre-flowering stage, five samples were collected from seeds, intact roots and shoots. The entire sampling was repeated three times independently. Samples were surface sterilized according to a standard protocol [2]. Sterilized tissues were ground in LB liquid medium and the extracts were plated onto Potato Dextrose Agar (PDA). Fungi with unique morphology were collected and re-cultured on fresh media for purity.

The *F.* *graminearum* strain used in this study (15 Acetyl DON Producer) was obtained from the Agriculture and Agrifood Canada Fungal Type Culture Collection (AAFC Food Research Centre, Guelph, ON, Canada). The effect of the crude culture extract of each fungus on the growth of *F.* *graminearum* was previously shown (Figure 1) [2].

### 4.2. Competitive Inhibition Experiment

To test the ability of each endophytic strain to competitively inhibit the growth of *F.* *graminearum*, co-incubation experiments were conducted. Each fungus was grown on PDA plates at 25 °C for one week. Thereafter, 5 mm agar plugs from each endophytic fungus were inoculated near the edge of fresh PDA plates, and 5 mm agar plugs of *F.* *graminearum* were inoculated on the other edge. Both *F.* *graminearum* and each endophytic fungus were co-incubated at 25 °C for two weeks. There were three replicates for each endophytic strain.

### 4.3. Plant Pathogenicity Assay

To confirm that the putative fungal endophytes are not pathogens, a plant pathogenesis assay was conducted. Seeds of finger millet were surface sterilized by washing in 0.1% Triton X-100 detergent (10 min), followed by 3% sodium hypochlorite (20 min, twice). Seeds were planted on sterile Phytagel based medium consisted of: 1 package Murashige and Skoog modified basal salt mixture (Catalog #M571, PhytoTechnology Laboratories, Shawnee Mission, KS, USA), 4 g Phytagel, 1 mL pyridoxine HCl (0.5 mg/mL), 500 µL nicotinic acid (1 mg/mL), 0.332 g CaCl_2_, 1 mL glycine (2 mg/mL), 10 mL thiamine HCl (100 mg/L), and 1 mL MgSO_4_ (18 g/100 mL), per liter. The medium was distributed into sterile glass tubes. Seven seeds were transferred to each tube and allowed to germinate in the dark for seven days, then transferred to light shelves (25 °C, 16 h light). When seedlings were one week old, each endophyte (or control) was applied onto the gel surface (11 mm diameter agar discs), in triplicate. Seedlings that were not inoculated with any fungi served as the negative control. Seedlings that were infected with the fungal pathogen *Alternaria* *alternata* were the positive control [43]. Plants were then assessed visually for disease symptoms at 10 days after inoculation with each endophyte or control pathogen. Then, the percentage surface area that showed lesions was quantified using Assess Software (Version 2.0, American Phytopathological Society, St. Paul, MN, USA) in comparison to controls. Results were statistically analyzed using Prism software version 5 (GraphPad Software Inc., La Jolla, CA, USA).

### 4.4. Root Colonization Assay

To test if the putative endophytes are able to colonize the roots of finger millet, confocal microscopy imaging was conducted. Finger millet seeds were surface sterilized and planted in glass tubes containing sterile Phytagel based medium (as described above). Each fungal endophyte was applied, in triplicate (100 μL of a 48 h old culture grown in potato dextrose medium) to finger millet seedlings (17 days after germination) and co-incubated with the seedlings at room temperature. The control consisted of finger millet seedlings incubated with potato dextrose medium only. Calcofluor white stain was used to stain fungi (catalogue #18909, Sigma Aldrich, St. Louis, MO, USA), following the manufacturer’s protocol. Thereafter, finger millet roots were scanned with a Leica TCS SP5 confocal laser scanning microscope (Leica Microsystems, Wetzlar, Germany) at the Molecular and Cellular Imaging Facility, University of Guelph.

### 4.5. Bio-Guided Purification of Active Anti-Fusarium Compounds

For large scale fermentation, each endophyte was fermented in 2 L flasks (five flasks per each endophyte) containing 50 g white rice and 100 mL H_2_O. The flasks were incubated at 25 °C for 30 days without shaking (Figure 1f–h). Then, each culture was extracted with ethyl acetate, and washed with water to remove salts and sugars. The extract was dried under vacuum at 45 °C and subjected to fractionation between methanol and *n*-hexane phases to remove long chain fatty acids. To purify the active compounds, the dry residues were separated by flash liquid chromatography. The column was filled with flash grade silica gel (SiliaFlash^®^ P60 230-400, SiliCycle, Quebec City, QC, Canada, Catalog # R12030B) and saturated with the desired mobile phase just prior to sample loading. For each endophyte, 3 g of the dry residue extract was dissolved in chloroform, mixed with silica and evaporated under vacuum. The extract was applied as a dried silica band on top of the column. The mobile phase (gradient combination of hexane/ethyl acetate followed by ethyl acetate/methanol) was then passed through the column under air pressure. Thin layer chromatography (TLC) was used to visualize the bands using different solvent-system combinations and vanillin-H_2_SO_4_ reagent. Eluted fractions were tested for anti-*Fusarium* activity using the dual culture agar diffusion method (as described below). Candidate fractions that inhibited the growth of *F.* *graminearum* were subjected to further purification using flash column chromatography and/or preparative TLC. Purified compounds were re-screened for inhibition of *F.* *graminearum* growth using disc diffusion assay. The active compounds were subjected to further spectroscopic structural elucidation.

### 4.6. Antifungal Assay of Purified Compounds Using Agar Diffusion Assay

To enable the bio-guided purification, the dual culture agar diffusion assay was employed. *F.* *graminearum* was grown for 48 h (25 °C, 100 rpm) in liquid potato dextrose broth (Catalog # P6685, Sigma Aldrich), then mycelia were added to melted and cooled PDA media (1 mL of fungal culture into 100 mL of media), mixed and poured into Petri dishes (100 mm × 15 mm), then allowed to re-solidify. Wells (11 mm diameter) were created in this pathogen-embedded agar by puncturing with sterile glass tubes, then a fraction/compound was applied into each well (100 µL per well) in triplicate. The agar plates were incubated at 25 °C for 48 h. The diameter of each zone of inhibition was measured (mm). Appropriate solvent mixtures were used as negative controls. Nystatin fungicide (Catalog #N6261, Sigma Aldrich) was used as a positive control at a concentration of 10 μg/mL.

### 4.7. Structure Elucidation of Active Anti-F. graminearum Compounds

Each purified compound was structurally elucidated using one and two dimensional nuclear magnetic resonance (NMR) techniques in combination with mass spectrometry (MS) methods and IR (infra-red) spectroscopy. NMR analyses were conducted at the University of Guelph-NMR Facility using a 600 DPX spectrometer (Bruker, Karlsuhe, Germany) operating at 600 MHz for ^1^H and 150 for ^13^C. Structural assignments were based on spectra resulting from the following NMR experiments: ^1^H, ^13^C, ^1^H-^1^H COSY, ^1^H-^13^C direct correlation (HSQC), ^1^H-^13^C long-range correlation (HMBC). IR spectroscopy was conducted using a Bruker Alpha IR Spectrometer instrument (Bruker, Karlsuhe, Germany) located in the Department of Chemistry, University of Guelph. MS was conducted in the Mass Spectroscopy Facility of the Advanced Analysis Centre of the University of Guelph using an Agilent LC-UHD Q-Tof (Agilent Technologies, Santa Clara, CA, USA) with the following acquisition parameters: Ion Source (ESI), Ion Polarity (Positive), capillary exit (Resolution, 140.0 V), Trap Drive (58.9 arbitrary units), Accumulation Time (1348 μs), Averages (3 Spectra).

### 4.8. Visualization of the Interactions Between F. graminearum and the Anti-F. graminearum Compounds

To visualize the interactions between *F.* *graminearum* and each pure anti-*F.* *graminearum* compound, light microscopy was used combined with vitality staining. Microscope slides were coated with a thin layer of PDA, then 100 µL of *F.* *graminearum* culture (48 h old grown in potato dextrose broth at 25 °C, with shaking at 100 rpm) were applied adjacent to 20 µL of each purified compound (5 mg/mL). There were three replicates for each slide. Slides were incubated at 25 °C for 24 h, stained with 100 µL of neutral red (Sigma Aldrich, Catalog #57993) for 3–5 min, then washed 3–4 times with de-ionized water. Images were taken using an MZ8 microscope (Leica Microsystems, Wetzlar, Germany).

## 5. Conclusions and Future Perspectives

A growing number of reports in the literature suggest that endophytic microbes may produce secondary metabolites that are also produced in parallel by their host plants [44,45,46]. Here, we reported the ability of endophytic fungi to produce small bioactive molecules previously reported as plant metabolites. It will be interesting to conduct future experiments to elucidate whether these apparent plant-derived compounds are in fact derived from their microbial inhabitants either exclusively or additively. It would be interesting to study the ability of each endophyte to inhibit the pathogen in finger millet and to test the pathogen specificity of each endophyte and its metabolite(s). As a general lesson, this study highlights the value of exploring the orphan crops of subsistence farmers as sources of endophytes and antifungal compounds with potential to combat serious pathogens afflicting mainstream, global agriculture.

## Supplementary Materials

Supplementary materials can be accessed at: http://www.mdpi.com/1420-3049/21/9/1171/s1.
