# Supplementary material for: Characterization of Antifungal Natural Products Isolated from Endophytic Fungi of Finger Millet (Eleusine coracana)
Source: Molecules. 2016 Sep 3;21(9):1171. doi: 10.3390/molecules21091171 (PMC6273740; doi:10.3390/molecules21091171)
Supplement: Supplementary file 1 [file molecules-21-01171-s001.pdf]

Supplementary Materials: Characterization of Antifungal Natural Products Isolated from Endophytic Fungi of Finger Millet (*Eleusine coracana*)

Walaa Kamel Mousa, Adrian L. Schwan and Manish N. Raizada

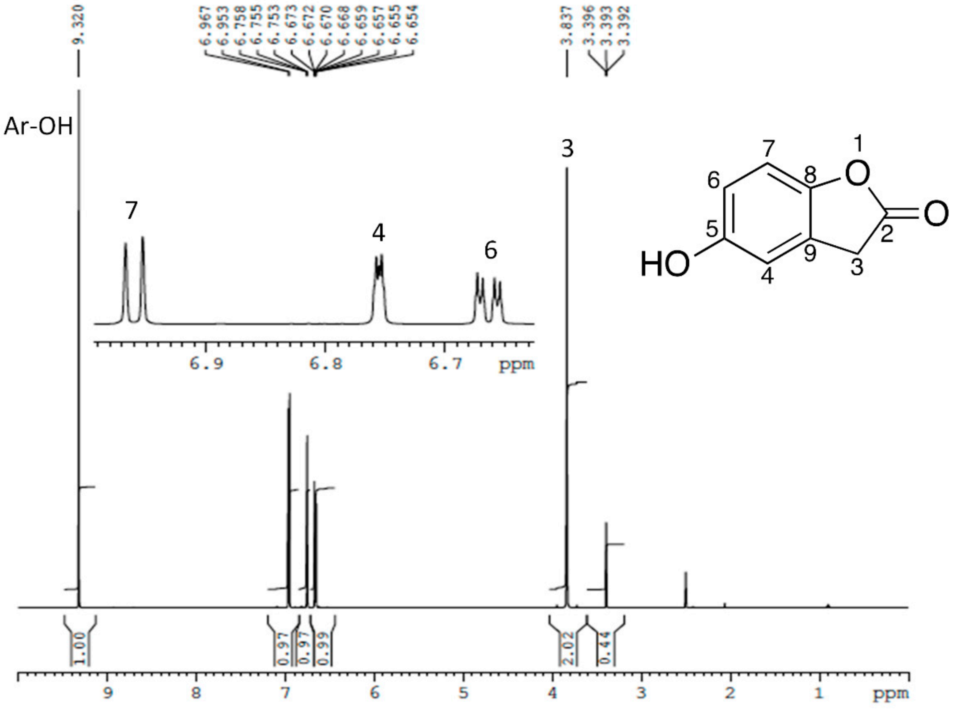

Figure S1. H[1]-NMR spectra of compound **1**.

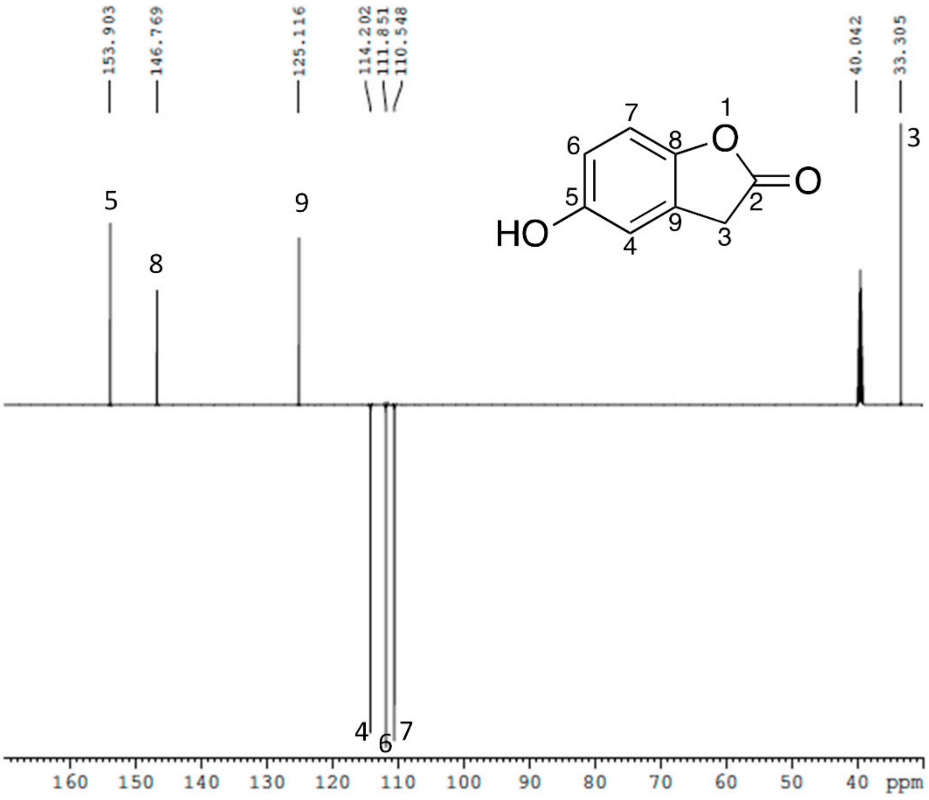

Figure S2. C[13]-NMR spectra of compound **1**.

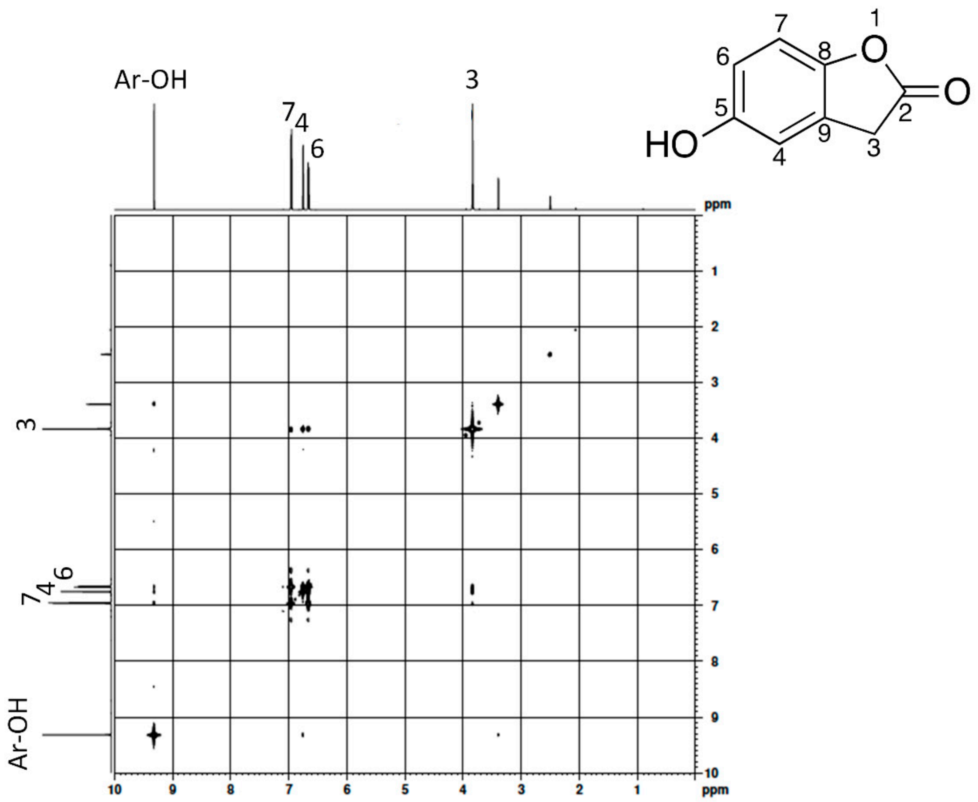

Figure S3. COSY spectra of compound **1**.

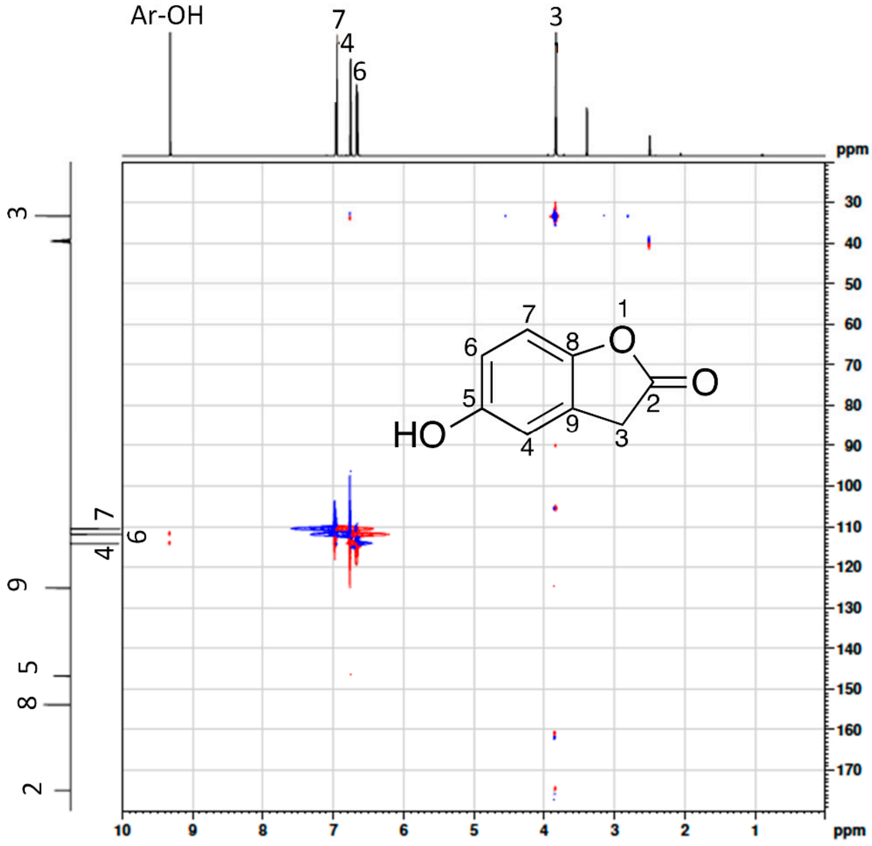

Figure S4. HSQC spectra of compound **1**.

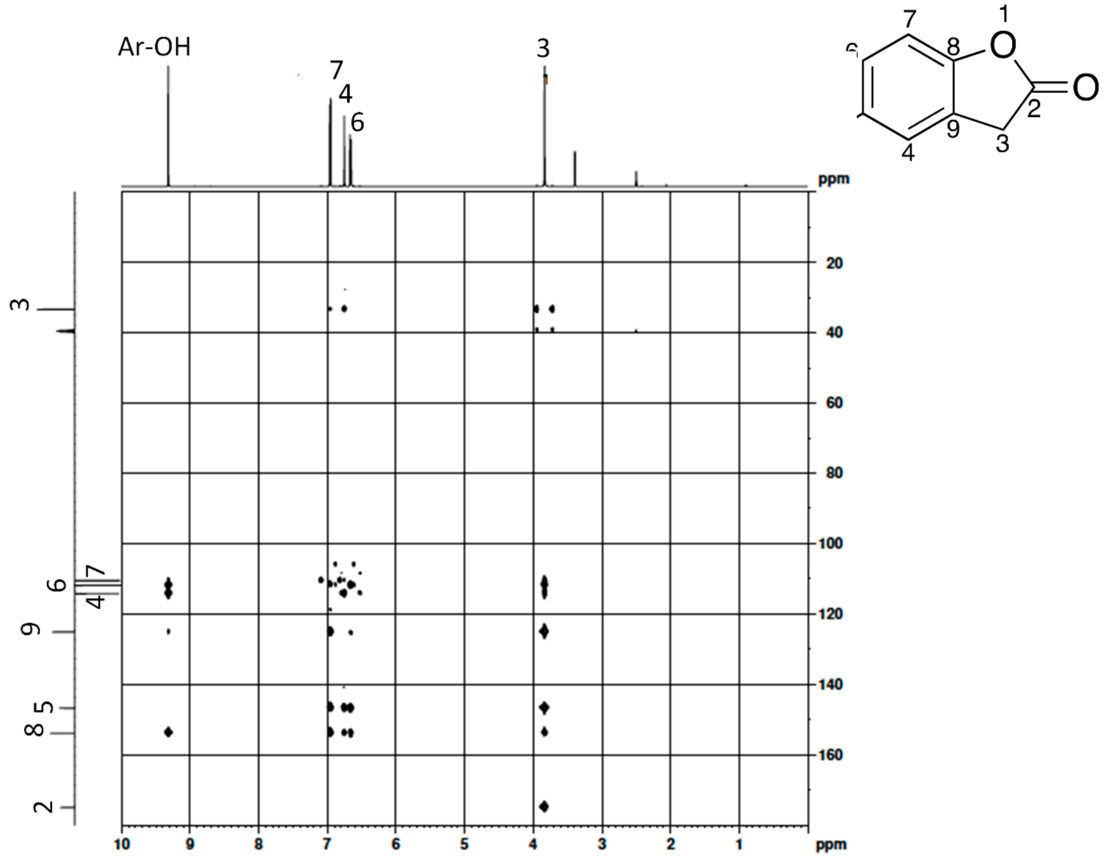

Figure S5. HMBC spectra of compound **1**.

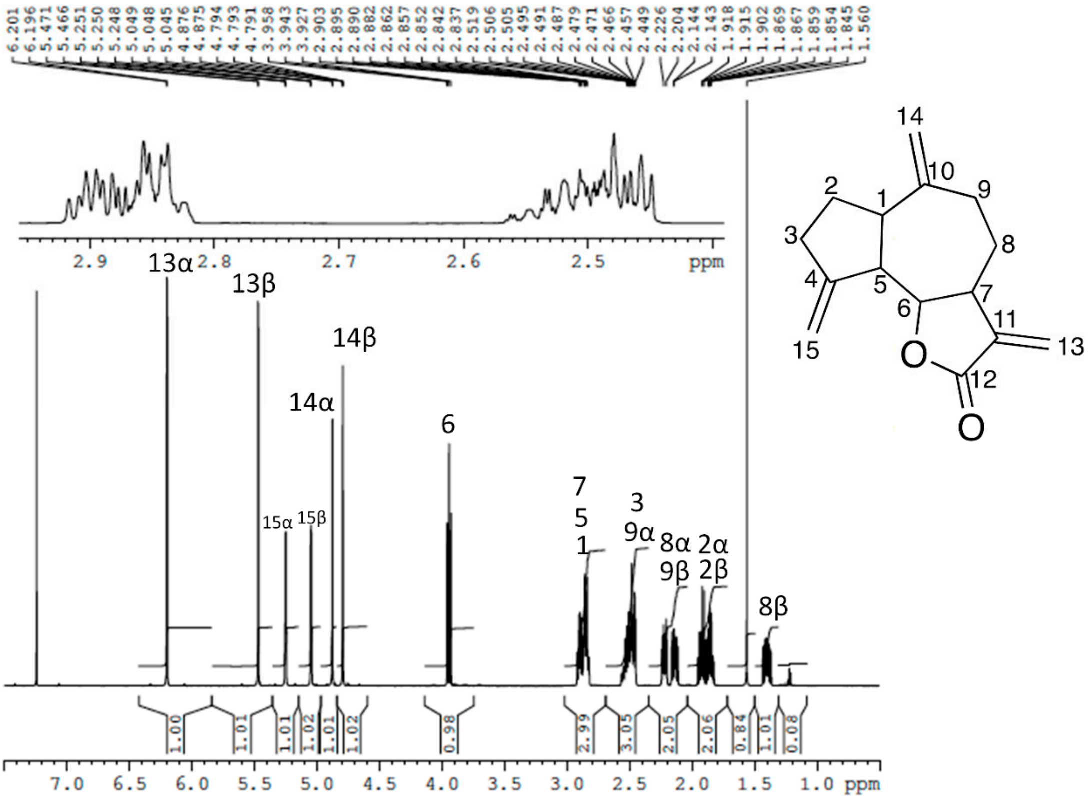

Figure S6. H[1]-NMR spectra of compound **2**.

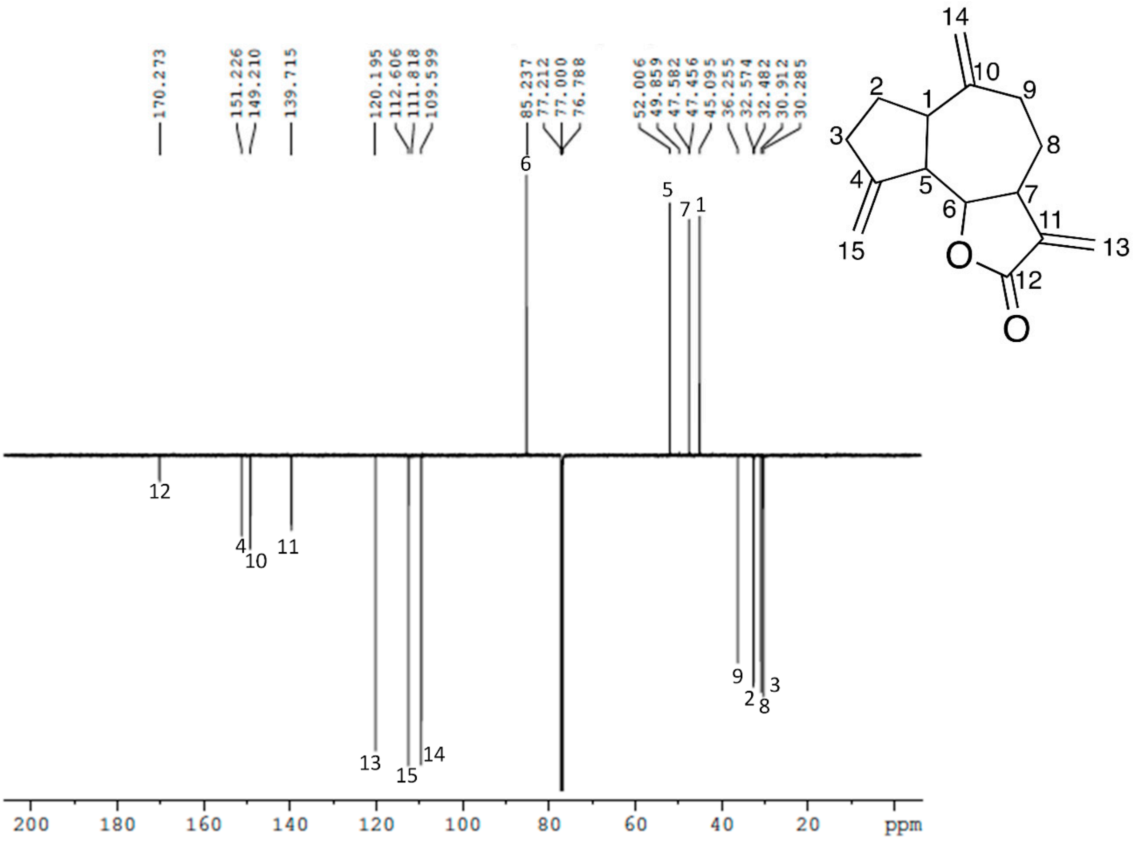

Figure S7. C[13]-NMR spectra of compound **2**.

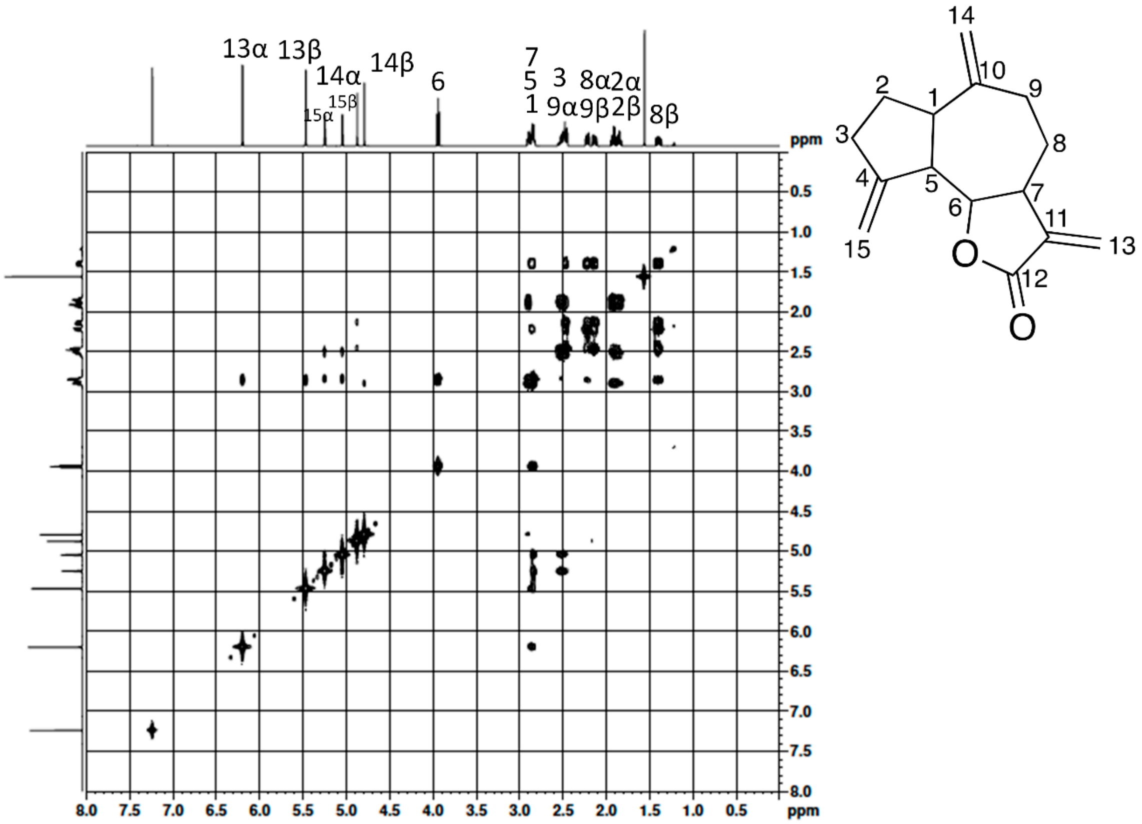

Figure S8. COSY spectra of compound **2**.

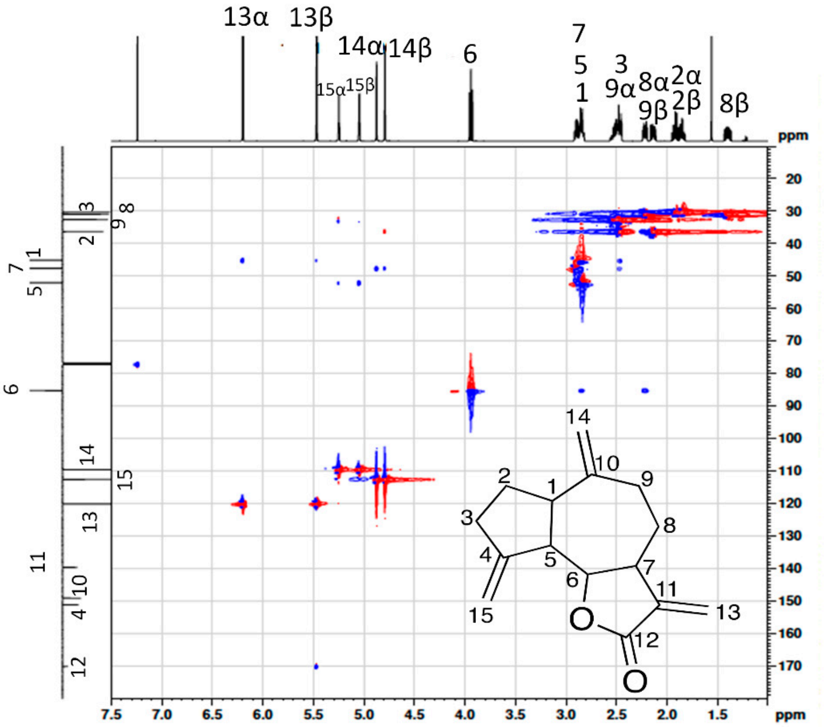

Figure S9. HSQC spectra of compound **2**.

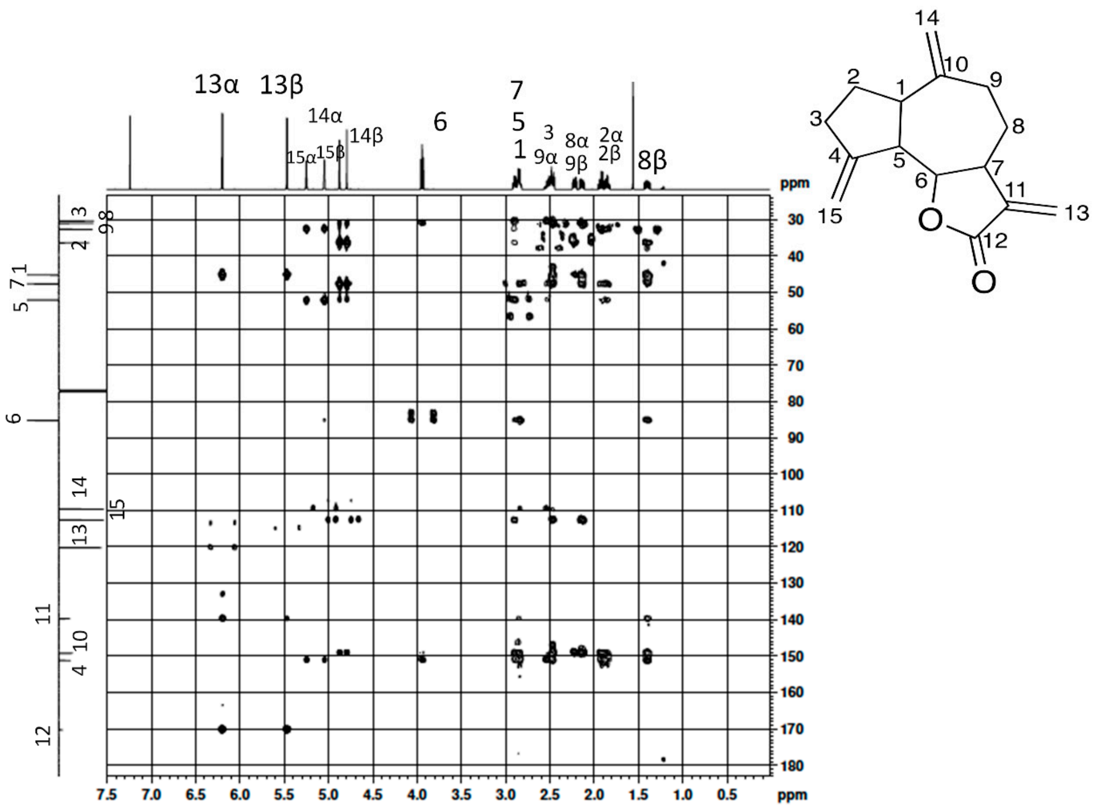

Figure S10. HMBC spectra of compound **2**.

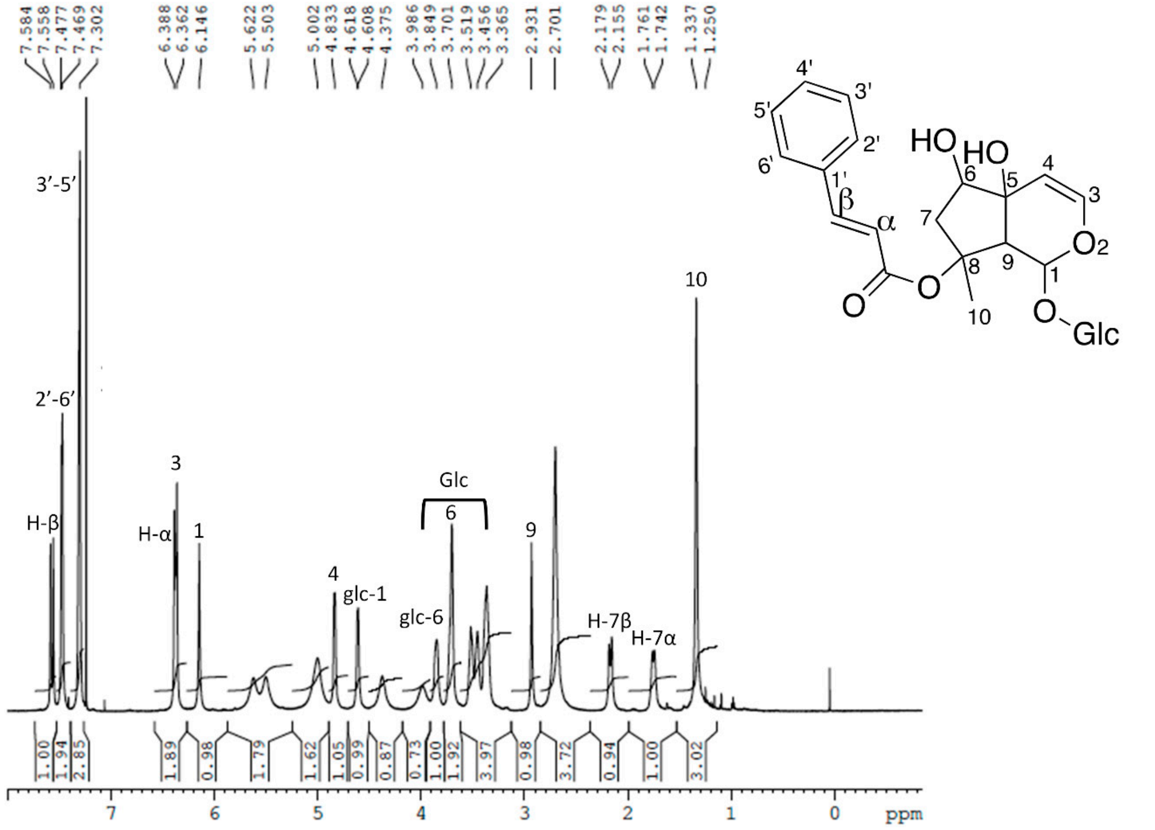

Figure S11. H[1]-NMR spectra of compound **3**.

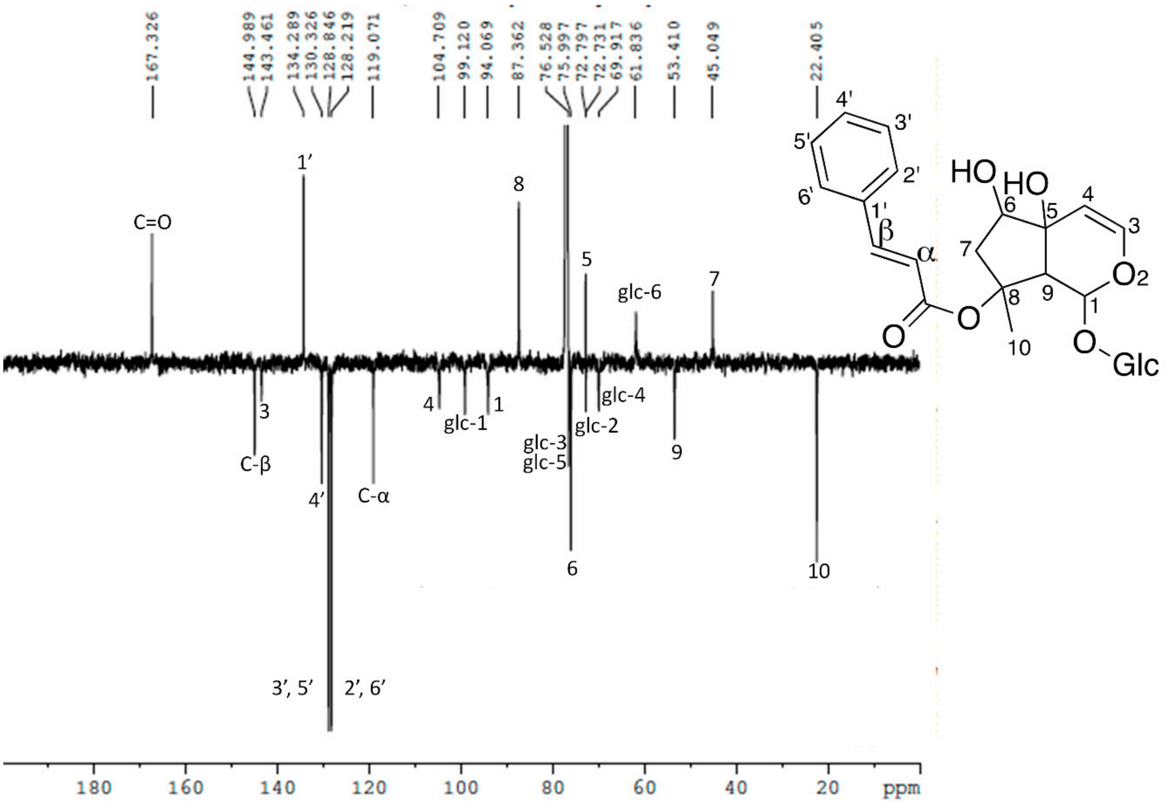

Figure S12. C[13]-NMR spectra of compound **3**.

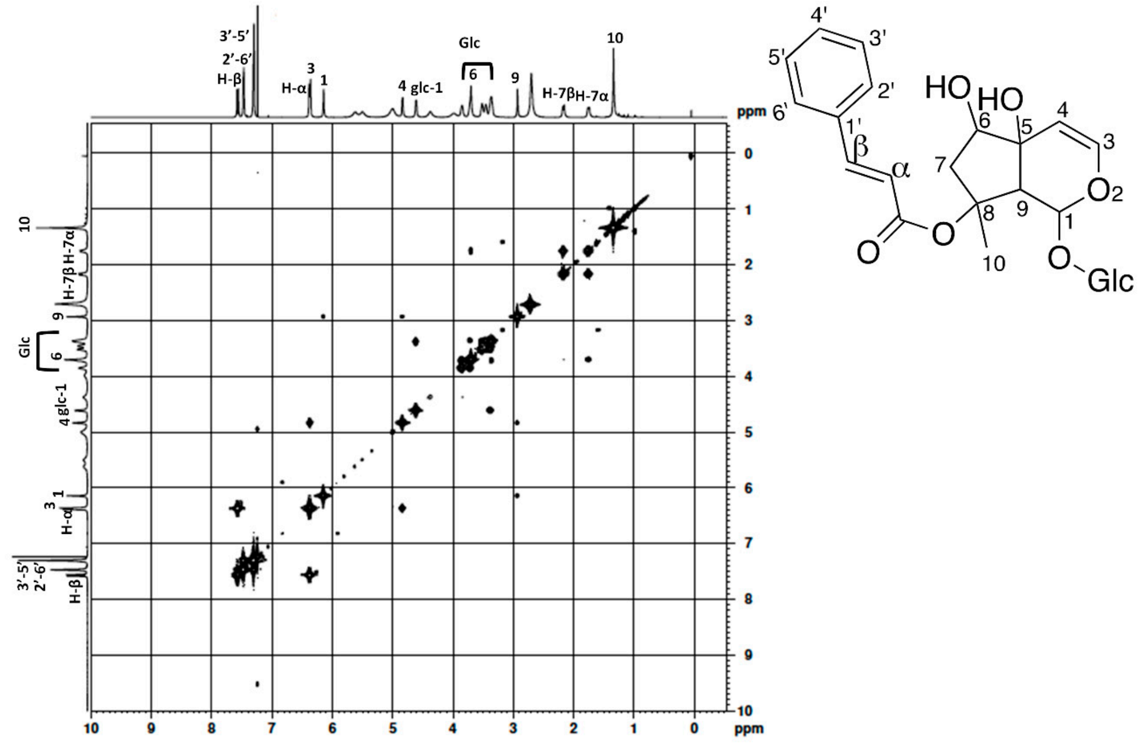

Figure S13. COSY spectra of compound **3**.

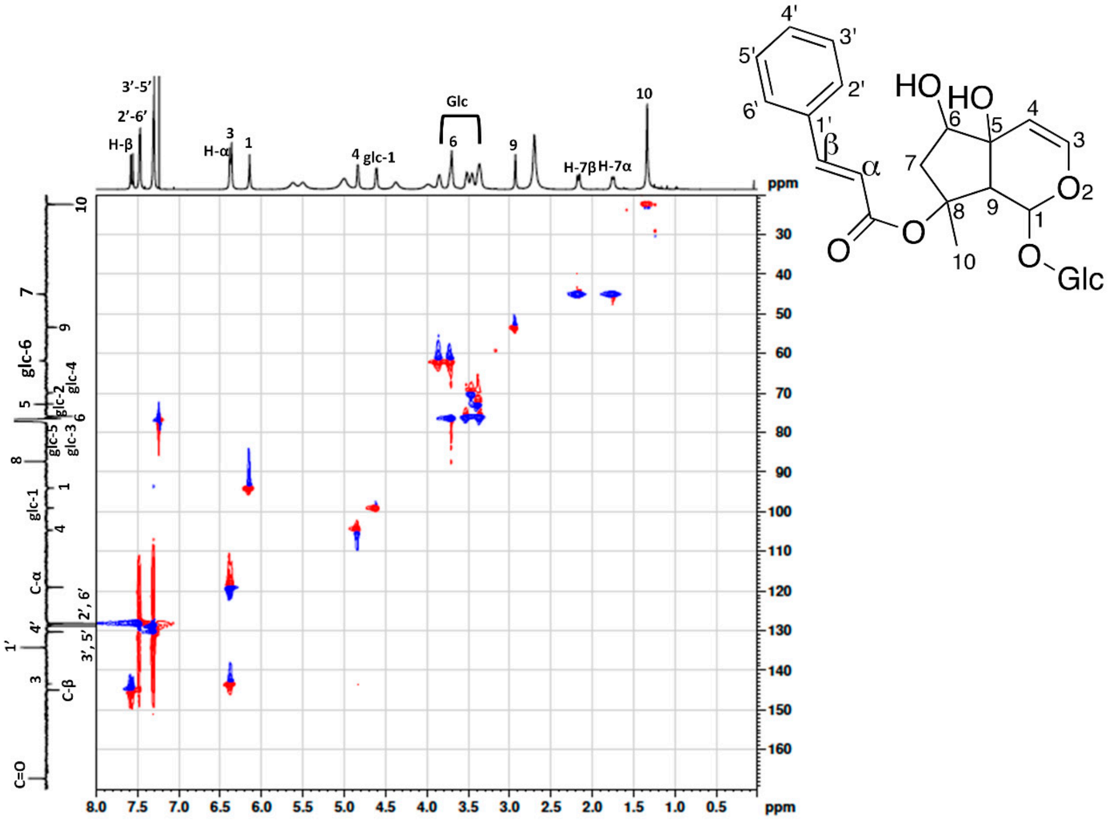

Figure S14. HSQC spectra of compound **3**.

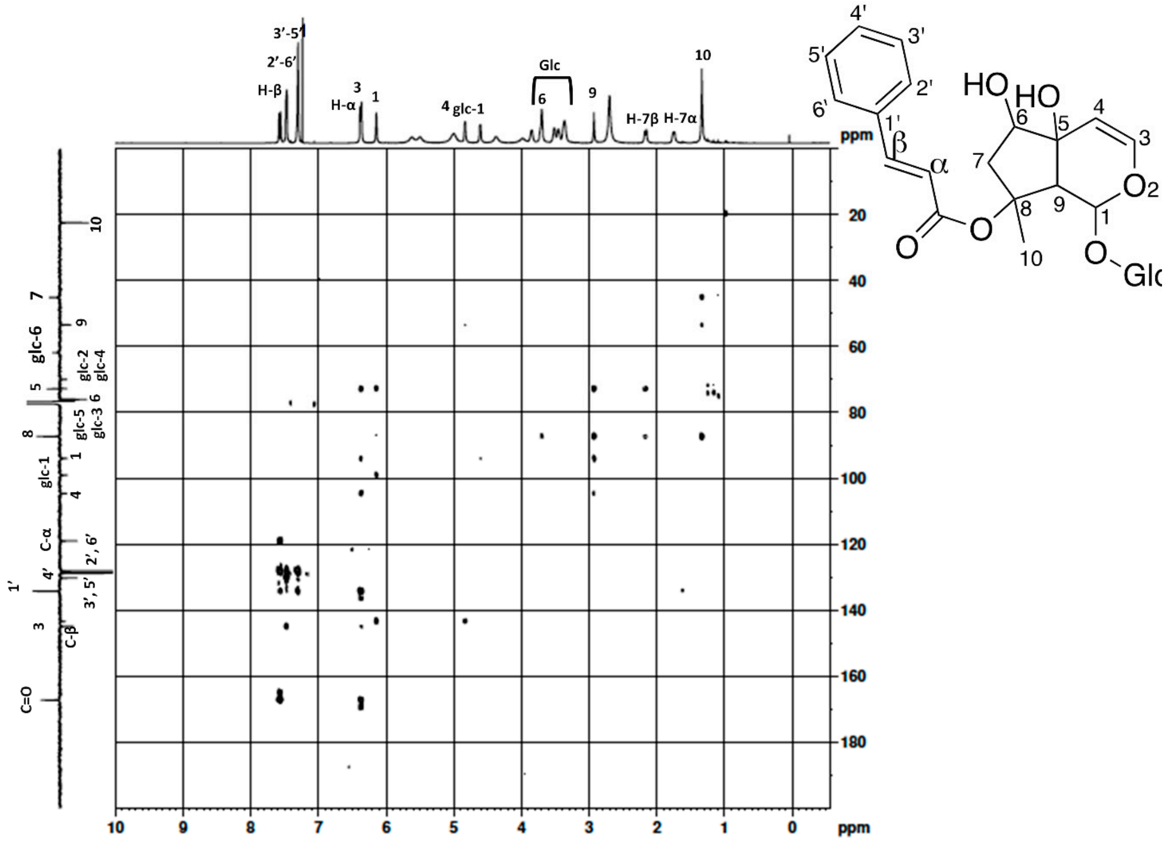

Figure S15. HMBC spectra of compound **3**.
